# Efficacy of EUS-guided keyhole biopsies in diagnosing subepithelial lesions of the upper gastrointestinal tract

**DOI:** 10.1055/a-2417-0580

**Published:** 2024-10-15

**Authors:** Sen Verhoeve, Cynthia Verloop, Marco Bruno, Valeska Terpstra, Lydi Van Driel, Lars Perk, Lieke Hol

**Affiliations:** 1 6993 Department of Gastroenterology and Hepatology, Erasmus Medical Center, Rotterdam, Netherlands; 2 7000 Department of Gastroenterology and Hepatology, Maasstad Hospital, Rotterdam, Netherlands; 3 2901 Department of Pathology, Medical Centre Haaglanden, Den Haag, Netherlands; 4 2901 Department of Gastroenterology and Hepatology, Medisch Centrum Haaglanden, Den Haag, Netherlands

**Keywords:** Endoscopic ultrasonography, Subepithelial lesions, Tissue diagnosis, Quality and logistical aspects, Performance and complications

## Abstract

**Background and study aims** Tissue acquisition is required for diagnosis of subepithelial lesions (SELs). However, obtaining adequate tissue remains challenging. This study investigated an EUS-guided technique using a forceps to create a channel and take multiple biopsies from the center of the lesion, therefore called endoscopic ultrasound-guided keyhole biopsy (EUS-KB).

**Patients and methods** A retrospective cohort study was conducted in 56 patients with SELs in the upper gastrointestinal tract who were scheduled to undergo EUS-KB. The primary aim was to assess diagnostic yield, defined as the percentage of procedures where EUS-KB resulted in a definitive histopathological diagnosis. Furthermore, factors influencing diagnostic yield were investigated. Additional outcomes included technical success and adverse events.

**Results** Technical success was achieved in 55 of 60 biopsies (91.7%). EUS-KB provided a diagnosis in 44 of 55 biopsies (80.0%), histology mostly showing gastrointestinal stromal tumor or leiomyoma. The diagnostic yield was not significantly influenced by the size or location of the SEL. Adverse events occurred in one patient (1.7%).

**Conclusions** EUS-KB is a feasible and safe technique for obtaining a classifying diagnosis for SELs in the upper gastrointestinal tract. It could offer an alternative diagnostic modality, especially in lesions smaller than 20 mm.

## Introduction

Expanding use of endoscopy has resulted in an increase in incidental findings of subepithelial lesions (SELs) throughout the digestive tract 1 2 . SELs are tumors located under the mucosa in the gastrointestinal wall and originate from one of the wall layers. The majority of SELs are benign and typically asymptomatic 3 . They encompass a wide variety of diagnoses including (pre)malignant lesions such as gastrointestinal stromal tumors (GISTs) or neuroendocrine tumors (NETs), as well as benign lesions including leiomyomas and ectopic pancreas 4 .

Diagnosing SELs may be challenging due to their subepithelial origin. There are various diagnostic modalities to identify potentially distinctive features of SELs. Endoscopic ultrasound (EUS) provides the most detailed morphological information, such as size, location, originating layer, echogenicity, and shape 5 6 . However, in only up to 64.2% of cases can EUS alone be used to establish the etiology of the SEL based on distinct pathognomonic imaging features 5 7 8 . Consequently, tissue sampling is required in the majority of cases to obtain a classifying diagnosis. The diagnostic yield of tissue sampling strongly depends on the acquisition technique used 5 . EUS-guided fine-needle biopsy (EUS-FNB) has been proven superior to EUS-guided fine-needle aspiration (EUS-FNA), with or without rapid on-site evaluation (ROSE) 9 10 11 . Recent systematic reviews reported equal efficacy of FNB and an emerging technique known as mucosal incision-assisted biopsy (MIAB) 12 13 . MIAB includes several approaches that can be performed during regular endoscopy. Recent studies suggest that deeper biopsy techniques such as MIAB may provide a higher diagnostic yield, particularly in lesions < 20 mm 14 15 . Therefore, the European Society of Gastrointestinal Endoscopy (ESGE) recommends using either MIAB or EUS-FNB for tissue diagnosis, with a preference for MIAB in lesions < 20 mm 5 . However, these recommendations are based on low-quality evidence. Newer deep biopsy approaches continue to be developed. One of these approaches includes EUS-guided keyhole biopsy (EUS-KB). The potential of EUS-guided forceps biopsies was highlighted in a small case series 16 .

The current study aimed to determine the diagnostic yield and safety of the newly proposed EUS-KB for diagnosing SELs in a large cohort of patients.

## Patients and methods

### Study design

A single-center, retrospective cohort study was conducted in the non-academic teaching hospital Haaglanden Medical Centre in The Hague, The Netherlands. The medical research ethics committee (MEC-U) and the local review board approved the study.

### Patients and data collection

All patients aged ≥ 18 years who were scheduled to undergo EUS with EUS-KB for diagnosing a SEL in the upper gastrointestinal tract between December 2013 and January 2023 were identified. All patients with intraluminal or extraluminal SELs of all sizes were eligible for EUS-KB. Informed consent was obtained from all patients before the procedure.

Patient demographics, lesion characteristics (e.g. size, location, echogenicity, originating layer, vascularity, and shape), procedure details (e.g. biopsy technique, use of hemoclips), procedure-related complications, histological diagnosis, and follow-up data including treatment were collected. Lesion size was defined by the maximum diameter measured during the EUS-procedure.

### Keyhole biopsy

EUS video of the forceps biopsy penetrating the submucosal lesion and taking multiple samples.Video 1

**Fig. 1:**
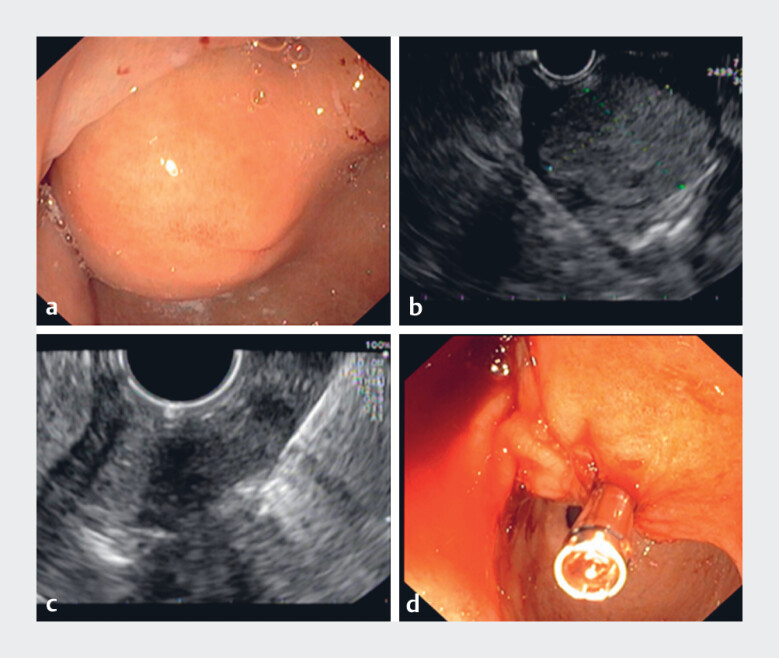
Visual overview of a EUS-KB intervention. **a** Endoscopic evaluation of the subepithelial lesion. **b** EUS evaluation of the subepithelial lesion. **c** After creating a channel trough the mucosa and submucosa, the biopsy forceps is introduced through the “keyhole” until it is certain that the tip is located within the SEL. Subsequently, multiple tissue samples are obtained from within the lesion. **d** The “keyhole” is closed with one or more clips.

EUS was performed in patients who mainly received conscious sedation using fentanyl and/or midazolam. EUS-KBs were obtained under EUS visualization by creating a channel into the mucosa and submucosa with use of a colon biopsy forceps (Radial Jaw 4 Biopsy Forceps, Boston Scientific) employing the so-called bite-on-bite technique. Once the wall of the SEL was penetrated, a cleansed biopsy forceps was introduced through the hole to obtain at least 10 samples from the center of the lesion ( Video 1 ). The mucosal incision was closed with clips with an EUS scope to prevent post-procedural bleeding ( Fig. 1 ). Biopsy samples were collected in formalin. The pathologists reviewed all samples and assessed the included layers and made a histological diagnosis; when applicable, additional microscopic determinations and immunohistochemical staining were performed.

### Outcome measures

The primary outcome was the diagnostic yield of EUS-KB, defined as the percentage of cases with a definite classifying diagnosis. Subsequently, additional analyses were performed investigating the diagnostic yield in relation to lesion size (< 20 mm and ≥ 20 mm) and location (esophagus, stomach, or duodenum).

**Fig. 2:**
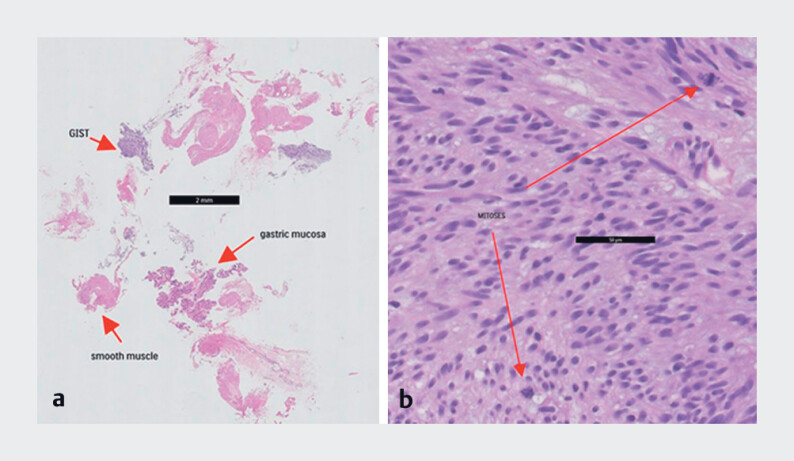
Microscopic view of a EUS-KB. **a** Hematoxylin and eosin-stained tissue sample acquired through EUS-KB. The sample shows a GIST. **b** A high power field for determining the mitotic index. This example shows two mitoses.

Secondary outcomes were technical success, tissue adequacy for risk assessment in GIST or NET, and adverse events (AEs) within 1 month related to the procedure. Complications were classified according to the AGREE classification 17 . Tissue adequacy was graded satisfactory with a mitotic count per 10 or 50 consecutive high-power fields (HPF) for NET and GIST, respectively. Mitotic count was defined as incomplete when less than 10 or 50 HPF could be analyzed. Fig. 2 **b** shows an example of a mitotic count for one HPF. The Ki-67 index was obtained for NETs.

### Statistical analysis

A descriptive analysis was performed to present multiple variables. Continuous baseline demographics were summarized with medians and interquartile range (IQR). Categorical variables were presented with frequencies and proportions. Categorical data between two groups were analyzed using Chi-squared test or Fisher’s exact test. Univariable logistic regression was utilized to identify the effect of lesions size or other predictors on a classifying diagnosis. *P* <0.05 was considered statistically significant.

Statistical analyses were performed in IBM SPSS Statistics for Windows, Version 28.0.1.0 (IBM Corp., Armonk, New York, United States).

## Results

### Baseline characteristics

In total, 57 consecutive patients with a SEL who were scheduled to undergo EUS-KB were identified. One patient was excluded because the pathology report was missing, resulting in 56 included patients. Four participants underwent an EUS-KB twice because the histology of the first attempt was inconclusive (n = 3) or the result was mistrusted was discounted by the endoscopist because of suspicious features on EUS (n = 1). Therefore, a total of 60 attempts with EUS-KB were included in the study.

The study population had a median age of 62 years (IQR 47–71). The median diameter of the SEL at first presentation was 20 mm (IQR 14.3–30). The SEL was symptomatic in nine patients (16.1%). SELs were mostly located in the stomach (n = 40; 71.4%). Twenty-seven patients (48.2%) had a biopsy preceding the EUS-KB, most of which were endoscopic biopsy techniques such as bite-on-bite biopsy. Baseline characteristics are summarized in Table 1 .

**Table:** **Table 1** Baseline characteristics.

**Parameter**			**Results**
Number of patients (n)			56
Number of procedures (n)			60
Age (median, IQR)			62 (47–71)
Female (n, %)			28 (50.0%)
Largest diameter SEL (mm; median, IQR)			25 (15–39.5)
Location	Esophagus (n, %)		11 (19.6%)
		Proximal	1 (1.8%)
		Mid	2 (3.6%)
		Distal	8 (14.3%)
	Stomach (n, %)		40 (71.4%)
		Cardia and fundus	11 (19.6%)
		Lesser curvature	7 (12.5%)
		Greater curvature	10 (17.9%)
		Antrum	12 (21.4%)
	Duodenum (n, %)		5 (8.9%)
		D1	3 (5.4%)
		D2	2 (3.6%)
Symptoms related to SEL			9 (16.1%)
	Gastrointestinal blood loss (n, %)		7 (12.5%)
	Weight loss (n, %)		2 (3.6%)
Previous attempt to obtain pathology			27 (48.2%)
	Endoscopic bite-on-bite (n, %)		10 (17.9%)
	Endoscopic other (n, %)		11 (19.6%)
	EUS-FNA (n, %)		2 (3.6%)
	EUS-FNB (n, %)		1 (1.8%)
	EUS-guided type unknown (n, %)		2 (3.6%)
	Bite-on-bite and EUS-guided (n, %)		1 (1.8%)
FNA, fine-needle aspiration; FNB, fine-needle biopsy; IQR, interquartile range; SEL, subepithelial lesion; EUS, endoscopic ultrasound.			

### Outcomes

#### Diagnostic yield

Biopsies were technically successful in 55 of 60 EUS-KB attempts (91.7%). Of the successful EUS-KB, 44 of 55 biopsies (80.0%) were diagnostic. Most lesions were GIST (n = 19; 34.5%), followed by leiomyoma (n = 16; 29.1%). Less frequently, the histology showed NET (n = 5; 9.1%) or lipoma (n = 2; 3.6%). The results are shown in Table 2 . Non-diagnostic EUS-KB was mostly due to an insufficient amount of submucosa in the specimen (9/55; 16.4%). Less frequently, the tissue was adequate for immunohistochemical staining; however, the final diagnosis remained unclear (2/55; 3.6%). When excluding second EUS-KB attempts, tissue sampling was diagnostic on the first attempt in 40 of 51 technically successful biopsies (78.4%). A second EUS-KB in four of these patients resulted in a histological diagnosis (GIST = 2; lipoma = 1) for three patients and it changed the diagnosis from leiomyoma to GIST for the other patient. In one of eight patients without a histological diagnosis after the first EUS-KB, subsequent EUS-FNA showed a schwannoma. Another patient was referred for surgical removal of the SEL; in that case, histology likewise showed a schwannoma. In five of eight patients without a diagnosis, follow-up was performed with endoscopy or EUS and new tissue sampling was omitted because the SEL had benign characteristics on follow-up. In one patient, for reasons unknown, follow-up never occurred.

**Table:** **Table 2** Diagnostic yield, technical success, tissue adequacy for risk assessment, and adverse events

**Histological diagnosis**			**N (%)**
Diagnostic yield			44/55 (80.0%)
Diagnosis	GIST		19/55 (34.5%)
	Leiomyoma		16/55 (29.1%)
	NET		5/55 (9.1%)
	Lipoma		2/55 (3.6%)
	Lymphoma		1/55 (1.8%)
	Inflammatory fibroid polyp		1/55 (1.8%)
Non-diagnostic			11/55 (20.0%)
	Insufficient amount of submucosa		9/55 (16.4%)
	Unclear diagnosis		2/55 (3.6%)
Tissue accuracy			18/19 (94.7%)
Tissue adequacy	GIST	Full mitotic count	9/19 (47.4%)
		Incomplete mitotic count	3/19 (15.8%)
	NET	Full mitotic count	2/5 (40%)
		Incomplete mitotic count	1/5 (20%)
Technical success			55/60 (91.7%)
Adverse events	AGREE llla		1/60 (1.7%)
GIST, gastrointestinal stromal tumor; NET, neuroendocrine tumor; AGREE, Adverse events in GastRointEstinal Endoscopy.			

Of the 43 patients with a classifying diagnosis, the SEL was surgically resected in 15 patients (GIST = 13, NET = 2). In one of these patients ultimately diagnosed with a GIST, a first biopsy attempt showed a false-positive result of leiomyoma, whereas the second biopsy correctly showed a GIST. All other surgical specimens were in accordance with the preceding biopsies. Endoscopic resection was performed in three patients with a diagnosis in accordance with the preceding biopsy (GIST = 1, NET = 2). This provided a tissue accuracy of 94.7% (18/19, Table 2 ). Surveillance was suggested in four patients with either EUS (GIST = 1; leiomyoma = 1), endoscopy (GIST = 1), or computed tomography scan (GIST = 1). Surveillance showed no growth in three patients (GIST = 2; leiomyoma = 1) and failed to occur in another patient (GIST = 1). No follow-up was advised in 18 patients because of a benign lesion (leiomyoma = 13; lipoma = 2; inflammatory fibroid polyp n = 1), in one patient for an unknown reason (GIST = 1), and in one patient because of comorbidities (NET = 1). One patient diagnosed with lymphoma was referred to the hematology department for management. Another patient was referred to an academic hospital because of obstructive complaints (leiomyoma = 1); further management details remain unknown. The management of one remaining patient is unclear; however, the GIST was not resected.

### Predictor analysis

As shown in Table 3 , the diagnostic yield of the first EUS-KB attempt was not affected by lesion size (76.0% for SELs < 20 mm and 77.8% for SELs ≥ 20 mm, *P* = 0.879) or SEL location (88.9% in the oesophagus, 71.1% in the stomach and 100% in de duodenum, *P* = 0.350). Additional univariable logistic regression showed no statistically meaningful predictors (age, gender, lesion size, location) affecting diagnostic yield.

**Table:** **Table 3** Relationship between diagnostic yield and lesion size and location.

**Variable**	**Diagnostic yield (n, %)**	**Result**
Lesion size	< 20 mm: 19/24 (79.2%)	X ^2^ (1)= 0.014, P = 0.904
	≥ 20 mm: 21/27 (77.8%)	
Location	Esophagus: 8/9 (88.9%)	*P* = 0.292
	Stomach: 27/37 (73.0%)	
	Duodenum: 5/5 (100%)	
*P* < 0.05 was considered significant.		

### Technical success

In five patients, the endoscopist was unable to perform an EUS-KB, resulting in a technical success rate of 91.7% (55/60). Reasons for technical failure were lesion mobility and small size of 5 mm (n = 1), a mobile lesion in the antrum of the stomach (n = 1), calcifications (n = 2), or a tough capsule (n = 1) making the SEL inaccessible with biopsy forceps.

### Tissue adequacy for risk assessment and accuracy

Tissue adequacy to estimate risk was assessed for specimens that showed a GIST or a NET (Table 2). It was possible to perform a full mitotic count in 50 HPF or 5 mm ^2^ for nine of 19 GISTs (47.4%). In addition, an incomplete mitotic count was obtained in three specimens that showed a GIST (15.8%). In the seven remaining GISTs, a mitotic count could not be performed. A full mitotic count per 10 HPF was achieved in two of five NETs (40%). In one NET, an incomplete mitotic count was obtained (20%). The Ki-67 proliferation index was determined in all NETs. Risk stratification changed in five SELs based on the mitotic count and Ki-67 index of the surgical specimen (GIST = 4; NET = 1). More detailed information about the mitotic count and risk stratification is provided in Supplementary Table 1.

### Adverse events

AEs occurred in one biopsy (1.7%, Table 2) in a patient finally diagnosed with an asymptomatic NET. The patient was readmitted to the hospital on the same day as the EUS-KB procedure due to hematemesis (AGREE llla). The patient used acetylsalicylic acid for cardiovascular prevention. A gastroscopy was performed to place an additional clip at the biopsy site, after which the bleeding stopped and the patient was discharged after 1 day.

## Discussion

Tissue acquisition is essential for diagnosing and managing SELs. However, obtaining an adequate specimen for diagnosis remains challenging 5 . The present study demonstrates the feasibility of a new EUS-guided biopsy technique (EUS-KB) with a technical success rate of 91.7% (55/60), a diagnostic yield of 80.0% (44/55), and a rate of only 1.7% major complications (1/60). There was no difference in diagnostic accuracy based on lesion size. Therefore, EUS-KB is a suitable and safe technique for tissue sampling of SELs (Box).

Current European guidelines suggest tissue sampling for SELs larger than 20 mm, high-risk stigmata, or when surgical or oncological treatment is required 5 . Recommendations for the type of tissue acquisition differ among guidelines, presumably because they are based on low-quality evidence 5 18 . Currently in Europe, MIAB or EUS-FNB are mostly recommended 5 . Conversely, American guidelines suggest using either EUS-FNA with ROSE or EUS-FNB as a first-choice modality 18 . Small lesions, in particular, remain more difficult to diagnose 14 19 20 . Newly developed techniques such as MIAB and other EUS-guided methods continue to emerge, with the aim of enhancing diagnostic capabilities 5 .

To date, only one case series of 10 patients has been published on EUS-guided forceps biopsy and reported a diagnostic yield of 100% without any AEs 16 . In the study, a needle knife was used to gain access rather than a biopsy forceps, as was done in our study. Due to a small sample size, superiority for either access technique cannot be determined.

EUS-guided forceps biopsies provide an alternative to the currently advised techniques of EUS-FNB or MIABA. A meta-analysis from Tan et al. reported a technical success rate of 98.8% and a diagnostic yield of 85.7% for EUS-FNB 21 . MIAB provided similar results compared with EUS-FNB, with a technical success rate of 95.7% and a diagnostic yield of approximately 88% to 91% 11 12 14 . These diagnostic yields are significantly higher than we found in our study (80.0%). However, EUS-FNB might be technically more challenging to perform on small lesions and as a consequence, lower diagnostic yields of 66% to 70% may be obtained 11 21 22 . Therefore, MIAB may be superior for diagnosing lesions smaller than 20 mm 12 14 . Our results also demonstrate a diagnostic yield of 76% in lesions smaller than 20 mm, significantly lower than the 93% for MIAB found by Minoda et al. although in agreement with the 80% found by Park et al 14 22 . The large heterogeneity found in MIAB studies can be attributed to a wide variety of biopsy techniques and study methods being used. An overview of the different techniques is depicted in Table 4 . Based on this study, EUS-KB might be suggested as an additional technique for lesions smaller than 20 mm, and as an alternative to the more challenging EUS-FNB in these type of lesions.

**Table:** **Table 4** Comparison of EUS-KB, EUS-FNB, and MIAB.

	**EUS-KB**	**EUS-FNB** 21 22 23	**MIAB** 12 14 23
Technical success	91.7%	98.8%	95.7%
Diagnostic yield	80.0%	85.7%	88%-91%
Small lesions	76%	66–70%	80%-93%
Adverse events	1.7%	1.26%	0%-7.5%
EUS, endoscopic ultrasound; KB, keyhole biopsy; FNB, fine-needle biopsy; MIAB, mucosal incision-assisted biopsy.			

The diagnostic yield in this study is slightly lower than has been reported for MIAB and EUS-FNB, but in the literature, various definitions of diagnostic yield have been applied, based on integration of immunohistochemical staining. Immunohistochemical staining is an essential element for diagnosing SELs. In this study, immunohistochemical staining was possible in all our diagnostic samples, compared with 46% to 69% in other studies 10 22 24 . In addition, several studies investigating EUS-FNB only included lesions larger than 15 mm or very few small lesions, and therefore, excluded possibly difficult-to-diagnose lesions 21 24 . Our study contained 14 lesions smaller than 15 mm (25%), possibly contributing to the lower diagnostic yield compared with EUS-FNB.

Assessing the mitotic count proved to be difficult. Specimens from EUS-FNB provide too little material for a complete mitotic count 25 . MIAB shows varying results with the possibility of a mitotic count in 22% to 100% of GIST specimens 26 27 28 . Often, a poor correlation exists between the mitotic count of a GIST in a biopsy and the surgical specimen 22 25 26 . EUS-KB might provide the possibility of a mitotic count in a substantial portion of the specimens. However, the consistency with the surgical specimens was poor. Therefore, biopsies might be unsuitable for risk assessment, but they aid in achieving a diagnosis.

The AE rate for all previously mentioned techniques was low 11 12 16 29 . Many MIAB studies reported no AEs; however, the rate may be up to 7.5% for the single-incision with needle knife biopsies (SINK), specifically 15 20 22 30 31 32 . For EUS-FNB, the complication rate was 1.26% 21 . AEs mainly consisted of bleeding, usually managed during the same procedure as the biopsy. EUS-KB, therefore, seems just as safe as EUS-FNB and slightly more safe than MIAB.

The success of a biopsy depends on the endoscopist’s experience and SEL characteristics. In addition, the availability of materials needs to be considered when choosing the right modality. EUS-KB offers potential because it can be performed simultaneously with EUS characterization, eliminating an additional visit for the patient. Moreover, EUS-KB requires readily available, relatively inexpensive materials without the need for an on-site pathologist. Furthermore, the diagnostic yield is not significantly influenced by lesion size. Conversely, EUS-KB may not be appropriate for mobile lesions, especially in the fundus or the greater curvature side of the antrum. A disadvantage of deep biopsy techniques such as EUS-KB and MIAB is scarring, therefore possibly precluding future endoscopic treatment for esophageal lesions 11 . Advantages of MIAB include that it is technically easier to perform and it can be performed by clinicians who do not have expertise with EUS or ROSE 22 27 29 33 . Nevertheless, EUS is usually performed before MIAB to characterize the lesion or visualize vessels before biopsy 5 30 . Moreover, MIAB may not be the preferred modality for SELs with an extraluminal growth pattern because endoscopic visualization alone may not be adequate to characterize the lesion. In contrast, EUS-KB could offer an advantage in such cases, because EUS enables visualization of extraluminal growth, providing a safer biopsy alternative. Unfortunately, whether that is the case remains unclear because there were no extraluminal SELs in our study 8 33 . Furthermore, MIAB takes longer than EUS-guided techniques 12 14 34 . In our study, procedure time was not formally recorded.

This study has several limitations, including a limited sample size drawn from a single center and two endoscopists performing the biopsies. In addition, it was a retrospective study without a comparison to the current biopsy standard. Conversely, it is the largest study available to date pertaining to this novel EUS-guided biopsy method. A strength of the study is the fact that it includes a whole spectrum of SELs because all patients with SELs scheduled for EUS-KB were included, which lowers the risk of selection bias. Therefore, the results are likely applicable in a population-based setting.

## Conclusions

In conclusion, this is the largest study on EUS-KB demonstrating its feasibility and safety for diagnosing SELs of the upper gastrointestinal tract. EUS-KB could offer an alternative diagnostic modality, especially for lesions smaller than 20 mm or when a previous attempt failed to provide a diagnosis. Currently, no studies exist comparing EUS-KB with other techniques. Additional research, preferably prospective trials comparing EUS-KB with other techniques such as MIAB and EUS-FNB, is needed to further investigate this procedure.

## Notes

Tips for performing EUS-KB.

1. Use a biopsy forceps with a spike (e.g. Radial Jaw, Boston Scientific) to keep the forceps stable.
2. Only use EUS visualization when creating the channel to stay in the right position. Orientation can easily be lost when using endoscopic view.
3. Take time to create the channel. It might take up to 10 biopsies or more.
4. Cleanse the biopsy forceps after every three or four biopsies.
5. When reaching the center of the lesion, verify the position of the biopsy forceps. The forceps might appear to be in the lesion when it is positioned next to the lesion.

(EUS, endoscopic ultrasound; KB, keyhold biopsy.)

## Supplementary Material

Supplementary Material Zusatzmaterial
