# Supplementary material for: Efficacy of EUS-guided keyhole biopsies in diagnosing subepithelial lesions of the upper gastrointestinal tract
Source: Endosc Int Open. 2024 Oct 15;12(10):E1183–9. doi: 10.1055/a-2417-0580 (PMC11479790; doi:10.1055/a-2417-0580)
Supplement: Supplementary file 2 — Supplementary Material [file 10-1055-a-2417-0580_24171416.pdf.pdf]

# Supplementary material

**Supplementary Table 1** Tissue details for risk assessment of GIST and NET.

| | Biopsy specimen | | | | | Surgical specimen | | |
|---|---|---|---|---|---|---|---|---|
| **Diagnosis** | **Size (mm)** | **Mitotic count** | **Ki-67 index** | **Risk stratification** | **Management** | **Mitotic count** | **Ki-67 index** | **Risk stratification** |
| GIST | 28 | < 5 per 50 HPF | | Low | Surgical resection | 1 per 50 HPF | | Low |
| GIST | 30 | < 5 per 50 HPF | | Low | Surgical resection | 8 per 5 $mm^2$ | | *Intermediate** |
| GIST | 40 | < 5 per 50 HPF | | Low | Surgical resection | 10 per 50 HPF | | *Intermediate* |
| GIST | 17 | 0 in 50 HPF | | Very low | Follow-up with CT scan | | | |
| GIST | 21 | Not described | | Low | Follow-up with EUS | | | |
| GIST | 35 | 2 per 20 HPF | | Low | Surgical resection | 5 per 5 $mm^2$ | | Low |
| GIST | 21 | 2 per 5 $mm^2$ | | Low | Surgical resection | 4 per 5 $mm^2$ | | Low |
| GIST | 30 | 1 per 50 HPF | | Low | Surgical resection | 3 per 5 $mm^2$ | | Low |
| GIST | 15 | 1 per 50 HPF | | Very low | No resection[†] | | | Low |
| GIST | 40 | 1 per 5 $mm^2$ | | Low | Surgical resection | 3 per 5 $mm^2$ | | Low |
| GIST | 57 | < 5 per 50 HPF | | Intermediate | Surgical resection | 14 per 5 $mm^2$ | | *High* |
| GIST | 50 | > 5 per 40 HPF | | High | Surgical resection | 23 per 5 $mm^2$ | | High |
| GIST | 80 | 5 per 40 HPF | | High | Surgical resection | 1 per 5 $mm^2$ | | *Low*[‡] |
| GIST | 90 | Not possible | | Unknown | Surgical resection | Not described | | Unknown |
| GIST | 40 | Not possible | | Unknown | Surgical resection | 1 per 10 HPF | | Low |
| GIST | 15 | Not possible | | Unknown | Endoscopic resection | 1 per 50 HPF | | Very low |
| GIST | 30 | Not possible | | Unknown | Surgical resection | 3 per 5 $mm^2$ | | Low |
| GIST | 17 | Not possible | | Unknown | Follow-up with EUS | | | |
| GIST | 25 | Not possible | | Unknown | No follow-up | | | |
| NET | 13 | < 1 per 10 HPF | < 1% | Grade 1 | No follow-up[§] | | | |
| NET | 30 | 1 per 10 HPF | < 2% | Grade 1 | Surgical resection | 2 per 10 HPF | ± 5% | *Grade 2* |
| NET | 11 | Not described | ± 2% | Grade 1 | Endoscopic resection | 0 per 10 HPF | ± 1% | Grade 1 |
| NET | 9 | 0 in 5 HPF | 1-2% | Grade 1 | Endoscopic resection | Not described | | Grade 1 |
| NET | 25 | Not described | ± 2% | Grade 1-2 | Surgical resection | 2 per 10 HPF | 4-5% | Grade 2 |

Supplementary material

CT, computed tomography; EUS, endoscopic ultrasound; GIST, gastrointestinal stromal tumor; HPF, high-power field; NET, neuroendocrine tumor.
*Second GIST found during surgery.
†Referred to academic hospital, further management advise unknown.
‡Size of surgical specimen 38 mm.
§Due to old age and comorbidities.
